# Distance, Duration, and Velocity in Cycle Commuting: Analyses of Relations and Determinants of Velocity

**DOI:** 10.3390/ijerph14101166

**Published:** 2017-10-02

**Authors:** Peter Schantz

**Affiliations:** 1 Research Unit for Movement, Health and Environment, The Åstrand Laboratory, The Swedish School of Sport and Health Sciences, GIH, SE-114 86 Stockholm, Sweden; peter.schantz@gih.se; Tel.: +46-8-12053818; 2 Unit for Occupational and Environmental Medicine, Department of Public Health and Clinical Medicine, Umeå University, SE-901 87 Umeå, Sweden

**Keywords:** cycling, commuting, distance, duration, velocity, environment, sex, age, body weight, BMI

## Abstract

*Background*: The distance, duration, and velocity of cycling for transport purposes are used in health economic assessments, epidemiological studies, traffic modelling, and planning. It is therefore of value to determine relevant levels for them, and analyze how they relate, as well as to what extent other relevant variables may affect cycling velocities. 1661 cycle commuters (34% males) in Greater Stockholm, Sweden have been studied for that purpose. *Methods*: The participants were recruited with advertisements. They received questionnaires and individually adjusted maps to draw their normal cycling route. Route distances were measured by a criterion method. Age, sex, weight, height, and cycling durations to work were self-reported. The commuting routes were positioned in relation to inner urban and/or suburban–rural areas. Linear multiple regression analyses were used. *Results*: Cycling speeds were positively related to commuting distances or durations, being male, of younger age, having higher body weight but lower body mass index (BMI), and using the last digits 1–4 or 6–9 in duration reports (as compared to 0 and 5), as well as cycling in suburban (versus inner urban) areas. *Conclusions*: The study provides new knowledge about how distance and duration, as well as other factors, relate to the velocity of commuter cycling. It thereby enables the use of more appropriate input values in, for instance, health economic assessments and epidemiological health studies.

## 1. Introduction

Distance, duration, and speed are basic characteristics of mobility in every mode of transport. To describe them is of interest in and of itself, but also from other perspectives. They are, for instance, used in traffic modelling and planning, as well as in evaluations of environmental, health, and economic effects. Cycling and walking have been less studied in these respects than the motorised forms of transport. Still, these variables represent important inputs in, e.g., cost-benefit analyses and health economic assessment tools for walking and cycling (e.g., [1] (p. 85) and [2]), as well as in selecting the metabolic equivalent of task (MET) values [3] and the intensity category for epidemiological studies on the relation between physical activity and health. Thus, it is important to develop more knowledge about distance, duration, and velocity in walking and cycling, for instance determine relevant levels for them, analyze how they relate, and to what extent other relevant variables may affect them. Here, focus is on cycling.

A great variability in cycling velocities has been reported in the literature (*cf.* [4,5]). This may be due to, for instance, the methodology used, the different cycling purposes, distances, durations, and characteristics of the cyclists, and the environments in which they cycle. Thus, there is a need to distinguish within both methodological issues and conceivable determinants of cycling speed.

Distance is difficult to measure. Self-reports on distances are generally too long and wide-ranging [6,7,8]. This is also true for active commuters [9]. Furthermore, objective methods based on known origins and destinations for trips and route choice modelling with geographic information systems (GIS) or global positioning systems (GPS) have been shown to overestimate cycling distances by about 5–20% [9]. However, a method that has been shown to be valid for distance measurements in active commuting is to let the commuters draw the routes taken and measure them using valid distance measuring techniques [10].

It is also a challenge to obtain valid durations of trips. The phenomenon of a preferential rounding off to the last digit values of 0 and 5 in self-reported trip durations (e.g., 10 and 25 min) (*cf.* [11]) indicates problems with validity in and of itself. However, this could be coupled to true duration values being more or less symmetrically gathered around the last digits 0 and 5, respectively. But since most studies indicate that self-reported durations are longer than those derived from GPS (*cf.* [12]), a more reasonable interpretation is that there is a systematic over-reporting of durations associated with rounding off to the last digits 0 and 5. This has also been noted when cyclists’ trips were followed with cameras [13]. One’s behaviour when reporting durations might, however, differ depending on the reason for the trip. It is possible that active commuting represents a type of trip in which both departure and arrival times are better known, possibly leading to more accurate reports on duration. Furthermore, these behaviours are normally repeated many times over the year [14]. To the best of my knowledge, it has not been reported whether preferential rounding off of the last digits in duration reports to multiples of 5 occurs among habitual cycle commuters, and, if so, to what extent. This issue is therefore analysed in this study.

Given that multiples of 5 in stating durations is almost the rule in some transport contexts (*cf.* [11]), it is hypothesized that individuals reporting durations with the last digits 1–4 and 6–9 (e.g., 13 and 28 min) may present more correct values. There is also support for this [13]. The hypothesis will be further scrutinised here by checking whether there are systematic differences in the estimated velocity of cycling depending on the last digit category in self-reported durations.

It was recently noted that a group of commuter cyclists, with longer distances than another group, also had higher cycling velocities [14]. This opens up the possibility of there being a positive relation between cycling distance and speed. In fact, another report indicates that this may be the case, but it was based on a very small sample [5]. In order to further scrutinise this possible relationship, other factors that may affect cycling velocity need to be considered. The power output in cycling is linearly related to oxygen uptake (e.g., [15]) and, in competitive cycling, the maximal oxygen uptake is an important predictor of physical performance [16,17]. The levels of that measure are related to sex and age [18,19] and can be taken into account in this study. Furthermore, differences in body mass may affect cycling velocity in various ways [20]. One of them is related to the fact that cycling involves transport of both a body and a vehicle and that a given weight of a bicycle will lead to a greater relative added weight for a light person than for a heavy one, which favours the cycling velocity of heavier persons. A higher body weight and body mass index (BMI) will, on the other hand, act in the opposite direction through increased energy demands at a given workload and pedalling frequency [21,22]. Also, the environment cycled in might induce different cycling velocities due to differences in the levels of route environmental variables (e.g., [23]).

Given this background, the overall aim of this study was to analyse whether cycling velocity was related to route distance or self-reported duration, sex, age, body weight, BMI, the last digit category (1–4 and 6–9 vs. 0 and 5) in duration reports, and the cycling environment. The study was undertaken in a stepwise explorative fashion, and is based on 1661 male and female cycle commuters in the metropolitan area of Greater Stockholm, Sweden.

## 2. Materials and Methods

This study is part of a greater multidisciplinary research project, Physically Active Commuting in Greater Stockholm (PACS), at the Swedish School of Sport and Health Sciences, GIH, in Stockholm, Sweden [24]. In this study, only fully active cycling is analysed, i.e., no cycling with electrically assisted bicycles (e-bikes) is considered. Approval to conduct this study was obtained from the Ethics Committee North of the Karolinska Institute at the Karolinska Hospital (Dnr 03-637), Stockholm, Sweden, and the participants gave their written informed consent.

### 2.1. Participants

Advertisements in two morning newspapers in Stockholm (Dagens Nyheter and Svenska Dagbladet) in 2004 resulted in 2148 responders. No incentives were provided. The criteria for inclusion were: a minimum age of 20 years, residency in the County of Stockholm (except for the municipality of Norrtälje), and to, at least once a year, cycle or walk the whole way to one’s place of work or study. It was emphasised that also persons with very short commuting distances were welcome to participate.

### 2.2. Questionnaires, Administration, Response Rates, and Inclusion Criteria

The participants responded to a paper-based questionnaire created for the study: Physically Active Commuting in Greater Stockholm (PACS Q1). It was pre-tested on a small convenience sample of academic staff members as part of the development. PACS Q1 is self-administered, in Swedish, and contains 35 items, only a few of which are used in this study, namely: sex, height, weight, year of birth and cycle commuting durations to work.

Out of the original 2148 responders to the advertisement, 133 did not return the PACS Q1, giving a response rate of 93.8%. Twenty-one of the responders did not meet the inclusion criteria, 276 were single-mode pedestrians and therefore were not included in this study, and 57 were excluded due to missing values in at least one of the studied variables. This left 1661 cycle commuting participants for this study.

### 2.3. Maps and Route Distance Measurements

An individually adjusted map was prepared based on each respondent´s written home and place of work or study addresses. The map was a black and white copy of the maps in the official telephone directory of Stockholm (scale 1:25,000). In a few cases, however, the relevant area was not found in that collection of maps, so the national outdoor map (scale 1:50,000) was used instead. The respondents were given instructions on how to draw their most usual cycle commuting route to and from their place of work or study on the map. In case of two or more places of work or study, they were asked to pick the one they spent the most time at, and, in the case of equal time spent, to choose one of them. However, before filling in the routes, they were asked to commute along their route once, and to note the street names. Finally, in case their routes included places outside the printed street grid network, such as parkways or tunnels, the cyclists were asked to mark their routes with specific carefulness. Furthermore, the homes were marked with the capital letter B, and a small box marking indicated their places of work or study. For further descriptions of the method, see Schantz & Stigell [10].

The maps were used to measure route distances using a criterion method with high validity and reproducibility [10]. For that purpose, a digital curvimetric instrument (Run Mate Club, CST/Berger, Watseka, IL, USA) was used by a technical assistant in two consecutive distance measurements. A third measurement was undertaken in 151 cases when differences between the first two measurements were greater than 5.8 ± 3.3 mm (mean ± SD), corresponding to 145 ± 82.5 m in real distance. The mean value of the two closest values of the three was used.

### 2.4. Commuting Durations and Estimations of Cycling Velocity

Participants were asked to record the times for their commuting trips on a normal day and when no other errands were undertaken. They wrote down their commuting time to their place of work or study in hours and minutes. The cycling velocity was calculated as a function of the map-measured trip distances and the self-reported durations.

### 2.5. Localisation of Trip Origins and Destinations in Relation to Inner Urban and Suburban Areas

Perceived levels of route environmental variables differ in many cases between the inner urban and suburban areas [23], which may affect the velocity of cycling (see also the study area description below). A distinction was therefore made concerning where within the study area the cyclists’ routes were located. It was based on the postal area codes in the participants’ home addresses and places of work or study. In that way, the origins and destinations of the trips were categorised in relation to their being in the inner urban or the suburban areas.

### 2.6. Study Area

The study area is the County of Stockholm, Sweden, except for the municipality of Norrtälje. In it, cyclists represent about 5% of the commuting trips, and the share of car drivers is about 40% [25]. This metropolitan area, with about 1.9 million inhabitants at the time of the study, consists of inner urban, suburban, and rural areas. The latter two areas will be referred to in the following as ‘suburban areas’. The boundary between the inner urban and suburban areas is shown in Wahlgren et al. [26]. In the year of data collection for this study, about 285,000 people lived in the inner urban area [27]. It also had a high density of workplaces and employees and is socio-economically linked with the surrounding territory by, for instance, commuting. For a detailed environmental description of the study area, see Wahlgren and Schantz [23]. Here, the topography, urban form, and residential density are described since these variables can affect cycle commuting speeds.

The natural landscape in the region is basically rather flat. The road system includes, however, rather frequently smaller and gentle slopes, and occasionally also more demanding hills of up to about 15 m in height. The inner urban area is a predominantly built-up area, with areal blocks in a grid-like pattern and a high intersection density. The residential density was approximately 13,000 residents per square km in the inner urban area [27]. The suburban and rural areas contained a mixture of residential areas, smaller industrial areas, and managed forests, as well as agricultural land. The streets are not normally laid out in a grid-like pattern. Instead, the main roads often follow old road networks formed during the agricultural period of the landscape, and the density of intersections, as well as traffic regulatory signaling systems (traffic lights), is clearly lower than in the inner urban area. The residential density of the suburban parts of the study area is indicated by two representative examples: the southern and western suburbs of the Municipality of Stockholm with approximately 3500 and 2900 residents per square km, respectively [27].

### 2.7. Characteristics of the Participants

The characteristics of the participants with regard to age, height, weight, BMI, duration of cycling, distance, velocity, and cycling environment are described for the male and female commuter cyclists in Table 1.

### 2.8. Analytical Approach and Statistical Analyses

Questionnaire data were entered in the Statistical Package for the Social Sciences and analysed in version 24.0 (IBM SPSS Inc., Somers, NY, USA). Distributions were checked for normality with the Kolmogorov-Smirnov test, and, based on the outcomes and the issues addressed, the values are stated as median values and first–third quartile, or means and 80% confidence intervals. Expected and detected distributions of last digits in self-reported durations (0 and 5 versus 1–4 and 6–9) were evaluated using chi-square analyses for each sex. For evaluating the effect of the last digit category on the estimated cycling velocities, the individual duration values were grouped in consecutively numbered clusters based on whether the last digits were 1–4 (=cluster 1), 5 (=cluster 2), 6–9 (=cluster 3), and 10 (=cluster 4) and so forth. The first two clusters (1–4 and 5 min) were omitted in the analyses since there were few individuals in the first cluster, and stating trip durations using full-minute reports can represent greater relative errors in these first two clusters compared to those that follow thereafter. Given that the number of participants became less than 11 in duration clusters representing more than 50 min and durations with the last digits 1–4 or 6–9, only durations ≤50 min were used. After these analyses, the relations between age, body weight, BMI, and category of cycling environment were plotted for each sex as error bars for the different duration clusters. This was done to check for any systematic differences among the clusters in variables that could affect the cycling velocity. It provided motives for using multiple regression analyses.

Before they were applied, linearity between the outcome cycling velocity and predictors with continuous variables was checked using scatter dots and lines fitting the values. Interrelations between the continuous variables were assessed with Pearson’s correlation coefficient. The variables distance and duration were highly correlated (r = 0.89), but they were not included in the same multiple regression analysis. The correlation coefficients for the remainder of the predictors were, in absolute values, r ≤ 0.76, thus indicating no problems regarding multicollinearity. As a limit, we used r > 0.80 [28] (p. 175).

Linear multiple regression models were used, and the predictors were entered simultaneously as a group. All durations ≤50 min were used in these analyses. Cycling speed was the outcome in both models, whereas the predictors were distance (model 1) or duration (model 2), and sex (reference = females, 1 = males), age, body weight, body mass index, the categories of the last digit in the self-reported durations (dichotomous categorical responses: reference = last digits 0 or 5; 1 = last digits 1–4 or 6–9), as well as cycling environment (three mutually exclusive dummy categories: suburban; suburban and inner urban; and inner urban, with suburban as a reference) (both model 1 and 2). The variance inflation factor (VIF) was used to check multicollinearity. The VIFs (all values ≤4.67; mean, 2.02) indicated no problems. Possible extreme data cases were identified using Cook’s distance. No such cases were found in either one of the models (all values ≤0.018; mean, 0.0007). Thus, conditions for multiple regression analyses existed. The results are presented as unstandardised regression coefficients B, 95% confidence interval (CI), level of significance and partial correlations, as well as R-squared (R^2^) for the overall models. To indicate significance, a statistical level corresponding to at least *p* < 0.05 was used.

## 3. Results

### 3.1. The Relation between Distance and Duration for Commuter Cycling Velocity

**Figure 1:**
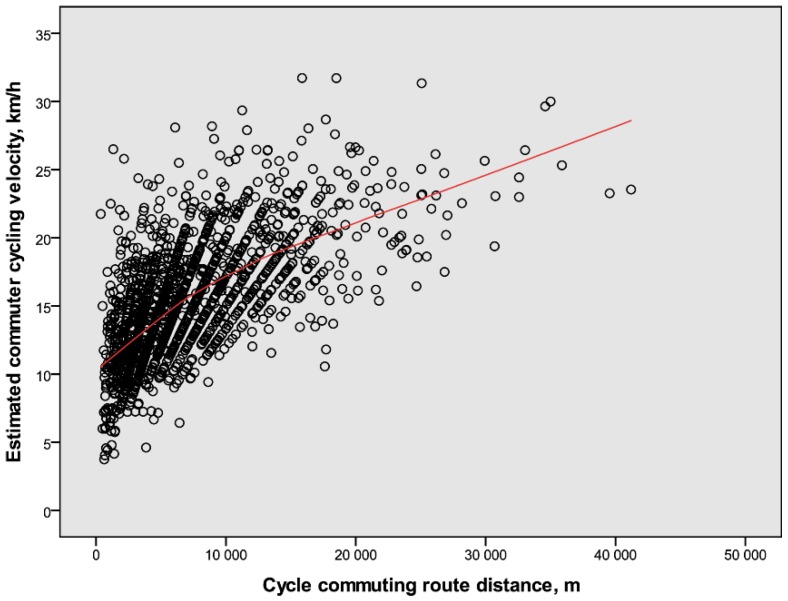
The relation between criterion-measured route distances and estimated velocities for commuter cyclists (*n* = 1661, 34% males). The line fits 85% of the individual values.

**Figure 2:**
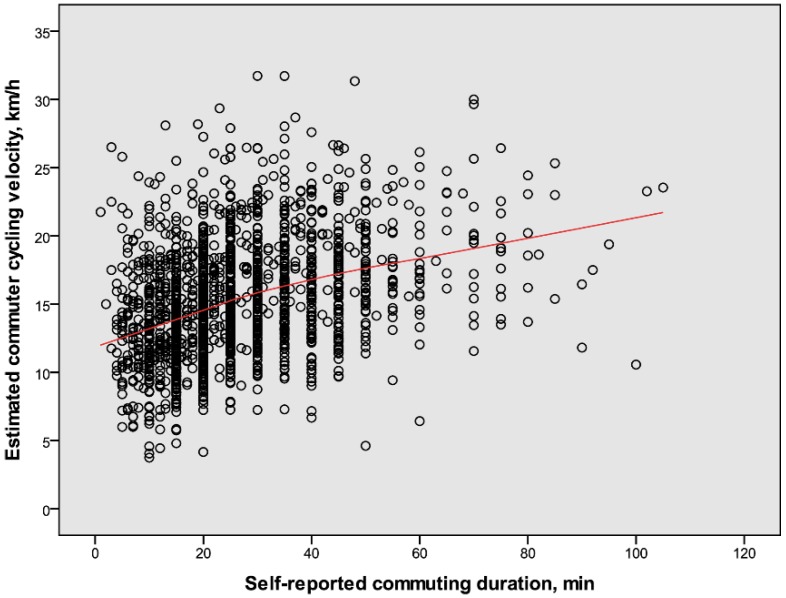
The relation between self-reported durations and estimated velocities for commuter cyclists (*n* = 1661, 34% males). The line fits 85% of the individual values.

The overall relations between distance and duration for commuter cycling velocity are illustrated in Figure 1 and Figure 2, respectively. Lines fitted to the values show that the estimated cycling velocities increased with both distance and duration. These positive relations appear to be steeper from low levels up to distances and durations of about 15 km and 45 min, respectively, whereas those after that are lower.

### 3.2. The Distribution of Self-Reported Cycling Durations

**Figure 3:**
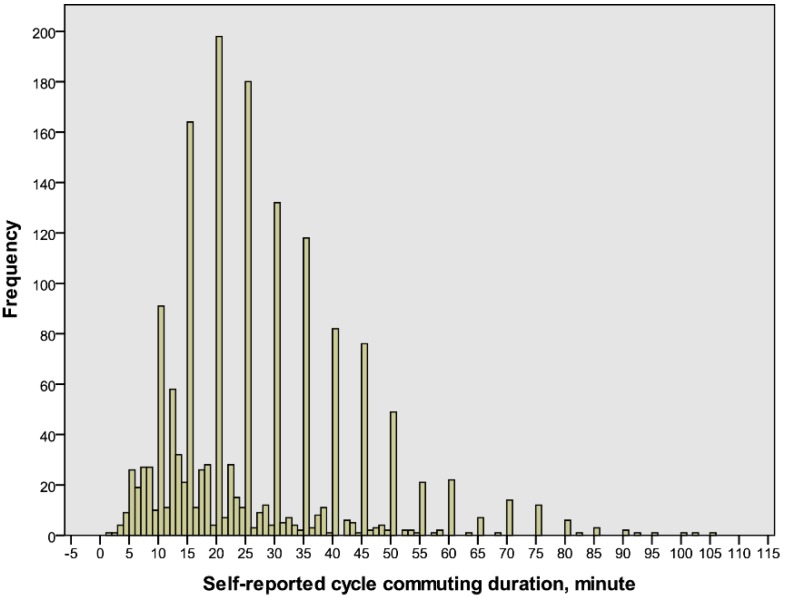
The frequency distribution of individual self-reported cycle commuting durations (*n* = 1661).

The accumulated number of self-reported commuting durations is illustrated in Figure 3. A preferential use of 0 and 5 as the last digits is evident. A comparison between the expected and detected proportions of the last digits 0 and 5 versus 1–4 and 6–9 reveal disproportionately high distributions of the last digits 0 and 5 in both males and females (*p* < 0.001) (Table 2).

**Figure 4:**
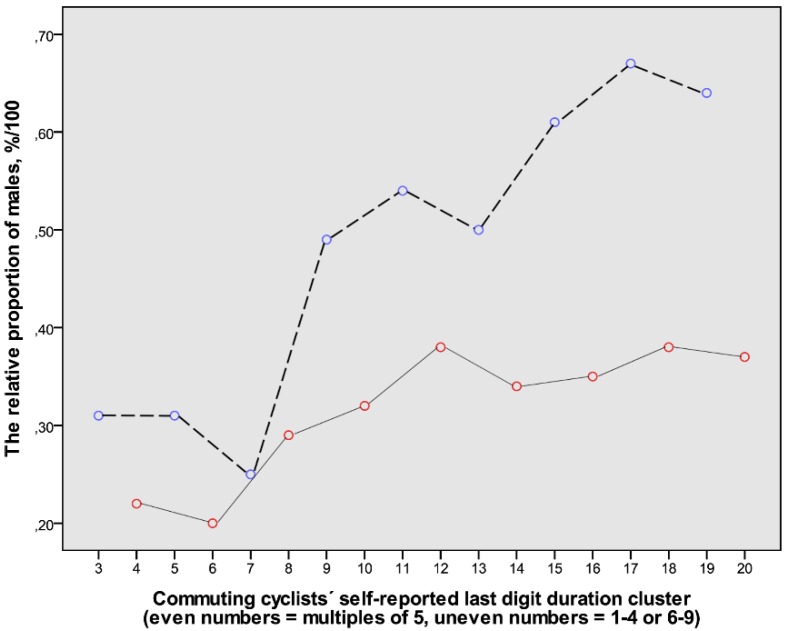
The relative proportions of data from male participants in clusters of self-reported cycling durations (*n* = 1516). The range of clusters (3–20) represents durations from 6–9 min to 50 min. The even-numbered clusters represent durations with the last digits being 0 or 5 (red symbols connected with continuous lines). The uneven-numbered clusters represent durations with the last digits being 1–4 or 6–9 (blue symbols connected with dashed lines). Cluster 4 = 10 min; 8 = 20 min; 12 = 30 min; 16 = 40 min; and 20 = 50 min. For further explanations, see Methods.

The duration values were divided into consecutively numbered clusters of last digit categories: cluster 1 = last digits 1–4; cluster 2 = 5; cluster 3 = 6–9; cluster 4 = 10; cluster 5 = 11–14, and so forth up to cluster 20, which represented the 50-min duration. The sex distribution was analysed in clusters 3–20 (Figure 4). It showed that males in general report durations with the last digits 1–4 and 6–9 to a greater extent than females, and that this is even clearer with increasing trip duration.

### 3.3. The Relation between the Self-Reported Duration Last Digit Category and the Estimated Cycling Velocities with Gender

**Figure 5:**
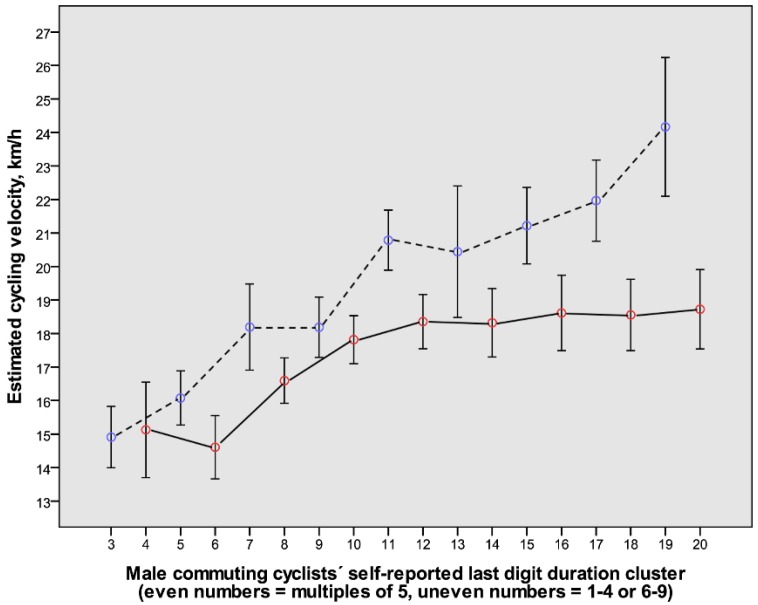
The relation between consecutive clusters of self-reported cycling durations and the estimated cycling velocities for males (mean value and 80% confidence interval) (*n* = 498). The even-numbered clusters represent durations with the last digits being 0 or 5 (red symbols connected with continuous lines). The uneven-numbered clusters represent durations with the last digits being 1–4 or 6–9 (blue symbols connected with dashed lines). For further explanations, see legend to Figure 4 and Methods.

**Figure 6:**
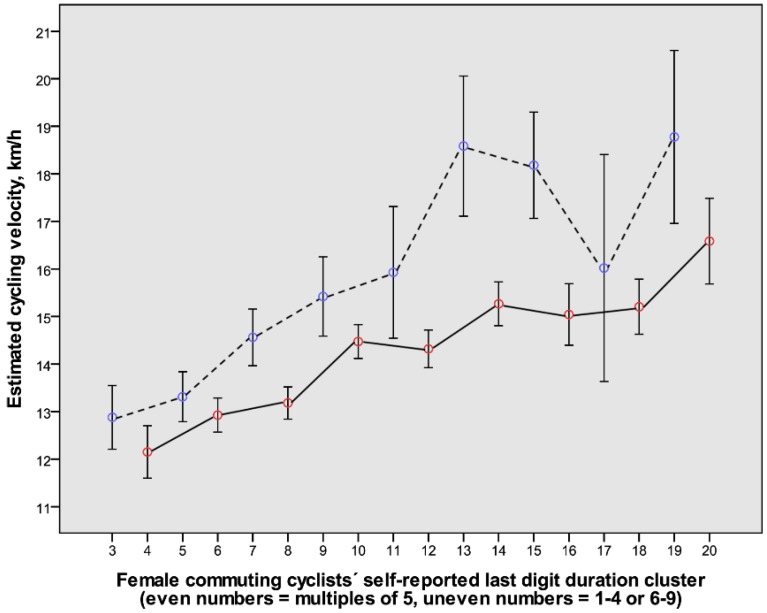
The relation between consecutive clusters of self-reported cycling durations and the estimated cycling velocities in females (mean value and 80% confidence interval) (*n* = 1018). The even-numbered clusters represent durations with the last digits being 0 or 5 (red symbols connected with continuous lines). The uneven-numbered clusters represent durations with the last digits being 1–4 or 6–9 (blue symbols connected with dashed lines). For further explanations, see legend to Figure 4 and Methods.

Given that the proportion of responses in last digit categories differed between the sexes (Figure 4), the analysis was furthered through sex-specific analyses. An overall trend towards higher estimated cycling speeds with longer commuting durations was noted in both sexes, as well as in the last digit duration clusters with 1–4 or 6–9, as compared to 0 or 5 (Figure 5 and Figure 6).

The relations illustrated in Figure 5 and Figure 6 motivated analyses to detect whether some conceivable determinants of cycling velocity altered in a systematic way with increased durations. The relations between age, body weight, BMI, and category of cycling environment were therefore plotted for each sex as error bars for the different duration clusters (data not shown). The levels of age, body weight, and BMI were stable with increasing duration categories in both sexes and therefore could neither explain the increased velocities with longer durations nor the variations between consecutive last digit duration categories. However, a slight but systematic difference in cycling environments was noted with increased durations, particularly in the males. Therefore, a natural next step to explore this complexity was to make use of multiple regression analyses.

### 3.4. The Relation between Cycling Velocity and Distance, as Well as Other Predictors

The relations illustrated in Figure 1, Figure 2, Figure 5 and Figure 6 motivated systematic analyses in the form of multiple regression analyses of possible determinants of cycling speeds. The correlations between continuous variables among outcome and predictor variables are presented in Table 3.

Multiple regression analyses revealed that cycling speeds were positively related to commuting distance, being male, of younger age, and higher body weight but lower BMI, and the last digits in self-reported durations being 1–4 or 6–9, as compared to 0 and 5, as well as cycling in suburban (versus inner urban) areas (Table 4). Based on the partial correlations, the most important variable for predicting cycling speed was route distance. The regression equation was: cycling velocity (km·h^−1^) = 16.2 + 0.64 × distance (km) + 1.69 × sex (1 = male) − 0.066 × age (years) + 0.036 × weight (kg) − 0.15 × body mass index (kg·m^−2^) − 1.66 × last digit in duration report (1 = 0 or 5) − 0.65 × cycling environment (1 = inner urban). For details, see Table 4.

### 3.5. The Relation between Cycling Velocity and Duration, as Well as Other Predictors

Cycling speeds were positively related to commuting durations, being male, younger age, higher body weight, lower BMI, last digits in self-reported durations being 1–4 or 6–9, as well as cycling in suburban areas (Table 5). Based on the partial correlations, three more important variables for predicting cycling speed were duration, sex, and age. The regression equation was: cycling velocity (km·h^−1^) = 18.3 + 0.096 × duration (min) + 2.67 × sex (1 = male) − 0.084 × age (years) + 0.051 × weight (kg) − 0.20 × body mass index (kg·m^−2^) − 1.49 × last digit in duration report (1 = 0 or 5) − 1.59 × cycling environment (1 = inner urban). For details, see Table 5.

## 4. Discussion

Important initial findings showed that commuter cycling velocities were positively related to both distance cycled and self-reported cycling durations. These results prompted to widen the analyses to also include other factors that could potentially explain the variations in velocity. This was undertaken in a stepwise exploratory fashion which will be commented upon below.

First of all, it was noted that self-reported cycle commuting durations are preferably stated as multiples of five, and that this is coupled with lower estimated cycling velocities compared to durations with the last digits being 1–4 or 6–9. This finding is in line with those of Kelly [13], who studied duration reporting in cyclists followed with cameras and found that over-reporting of durations was a general phenomenon, but that those choosing 0 and 5 as their last digits over-reported their durations to ~200% higher degree than those reporting last digit durations of 1–4 and 6–9.

Interestingly, independent of last digit duration category, cycling velocities increased in both males and females with increasing durations (Figure 5 and Figure 6), and when checked for, it was not apparent that it was due to other conceivable factors, such as a systematic difference in age with different durations. To fully check for this, multiple regression analyses were undertaken, and they revealed that cycling speeds were positively related to commuting distances or durations, being male, of younger age, having higher body weight but lower BMI, and self-reported durations with the last digits 1–4 or 6–9, as well as cycling in suburban areas. The findings will be discussed below.

The cycling velocity increased in the full models by about 0.64 km·h^−1^ per km of distance, and by about 0.10 km·h^−1^ per minute increase in duration. One consequence of this is that energy demands per unit of time of cycling, and thereby the metabolic equivalent of task (MET) values, increase with both durations and distances. This is relevant for consideration in, for instance, physiological and epidemiological studies, as well as in health economic assessments. For example, the WHO Health Economic Assessment Tool (HEAT) for Cycling [2] makes, at present, use of a fixed MET-value of 6.8 and a cycling velocity of 14 km·h^−1^ for both males and females independent of distance or duration. The findings in this study lend support to a development in that respect.

These relations were calculated on the basis of the duration range 1–50 min, and it is reasonable not to extrapolate the increase in velocities beyond 50 min or the corresponding distance. This is because analyses of the remainder of durations (not shown) indicated that there was no further significant increase in velocity with increased duration, or a clearly smaller increase was noted with increased distance.

The explanatory power with sex and age as predictors (Table 4 and Table 5) is in line with their relation to maximal oxygen uptake [18] and [19] (p. 306) and its relation to speed in competitive cycling [16,17]. Furthermore, the power output during cycling is related to levels of oxygen uptake [15].

Combining body weight and BMI as predictors provides an opportunity to evaluate the integrated approximate effect of body size and body fat. In a large group of slender children, youth, and young adults, the overall sex-neutral relation between body weight and maximal oxygen uptake indicates the same relative increases in these variables [29] (p. 106). Based on that, body weight could be expected not to be a relevant predictor of cycling speed. However, body mass may affect cycling velocity in various ways [20]. One rationale for including it here as a predictor is that cycling signifies transporting both a body and a vehicle. The most common weight of the male and female bicycles sold in the Stockholm region has been about 18.5 kg for a number of years (personal communication with bicycle dealers). Adding that weight will lead to ~30% increase in the body + bicycle weight of a 60-kg person, but only ~20% for a person weighing 85 kg. That speaks in favour of persons with greater body mass being able to cycle faster since the relative effect of the added cycling weight can affect both the rolling resistance and the gravitational effect on ascents [20]. Another aspect favouring taller persons, who generally weigh more, relates to the cyclist’s body surface area, which affects wind resistance. In relative terms, it is lower for a taller person [20]. On the other hand, a higher body weight and BMI will act in the opposite direction through increased energy demands at a given pedalling rate and workload [21,22]. Interestingly, the overall effect of body weight was positive for cycling velocity, whereas, as could be expected, BMI had a negative effect.

The behavioural dichotomy of duration reporting as a predictor in the multiple regression analyses shows that its effect (1.49–1.66 km·h^−1^ higher velocities with last digit durations of 1–4 and 6–9, as compared to last digit durations of 0 and 5) is generalised to different distances or durations, ages and sexes, etc. Against the background given above, the relation between speed and distance or duration reported by individuals with last digit duration reports of 1–4 and 6–9 is interpreted to be a more correct representation of the actual values, and it is recommended to be used in such applications as epidemiological studies, traffic modelling and planning, health economic assessments, and cost-benefit analyses.

Another novel finding is that the environment cycled in affects the velocities, with about 0.65 km·h^−1^ higher speeds being noted for a given distance cycled in the suburban areas as compared to the inner urban areas in a metropolitan setting. In light of the differences in environmental conditions between these areas [23], these results may not, however, be surprising.

A sensitivity analysis (not shown) of the two multiple regression models revealed that the variables distance or duration, age and sex predicted 85–95% of the variations in cycling speed, in comparison with the R^2^-values of the full model. Adding body weight and BMI as predictors had a very minor influence on those levels, but led to between 16–18% decreases in the size of the unstandardised regression coefficient B for sex. When omitting either BMI or body weight as predictors in the full regression models, the corresponding increases were 23% in regression coefficient for sex. It illustrates that those variables play a role in the overall sex differences noted in cycling speed.

The study is cross-sectional and therefore one cannot state anything for certain about the noted relations in terms of an intra-individual behavioural phenomenon. The increased speeds with distance and duration could, in principle, be due to a more or less instantaneous programming to higher cycling efforts when we have longer cycling distances ahead of us and/or a systematic selection of individuals with a greater capacity for higher cycling speeds involving themselves in longer cycling durations or distances. Another possibility is that it may be due to a conscious selection of lower speeds when distances or durations are shorter to avoid sweating. Indeed, intra-individual studies on speed selection with different distances or durations would be of great value to further our understanding of these issues. Based on the findings of this study and other ones, a strong recommendation for future studies and investigations of these matters is to inform the participants about the importance of accurate duration reports, which excludes rounding off to the last digits of 0 and 5. Use of the time measuring function in, for example, cell phones should be encouraged, and adding seconds in the questionnaire responses might possibly signal the importance of accurateness. It is, of course, also important to measure distances correctly, which is facilitated by open access geographic information systems available on the Internet, (e.g., gmap-pedometer; www.gmap-pedometer.com). GPS traces can nowadays be easily obtained by means of smart phones for collecting data on time, distance, and velocity, as well as the route area cycled in. Compensations for about 5% systematically longer distance measurements with GPS need to be considered, however [9]. If no use is made of such measuring steps, it is recommended to use stated addresses at the trips’ origin and destination points, and to measure the straight line distance between them and multiply it by 1.25 [9]. That will clearly provide more adequate distance data than asking participants about which route distances they believe they have cycled.

### Strengths and Limitations

A clear strength of the study is the number of cyclists involved, the variations in the values of all predictors, and that route distances were measured by a criterion method. Another strength was that sex, age, body weight, BMI, the last digit category in duration reports, and the cycling environment were included in the multiple regression analyses. A limitation was the preferential reporting of last digit cycling durations of 0 and 5, which adds uncertainty to the durations reported. The external validity of the findings should also be considered. Given that the sample is gathered from a small proportion (~5%) of the general population (*cf.* [25]), it is possible that the cycling velocities do not mirror the general population. Data were collected in September and October, and during that period it is light during the predominant commuting hours (*cf.* [14]). It is possible that the speed would be lower if cycling in the dark. Another aspect relates to the topography. A different degree of hilliness will most likely affect the cycling velocities. These types of matters deserve future studies.

## 5. Conclusions

This cross-sectional study shows that commuter cycling velocity is positively related to the distance and duration cycled. Sex and age are other important determinants, and adding body weight, BMI, and the last digit category in self-reported durations, as well as the area cycled in, creates even more correct input values for future epidemiological studies, traffic modelling and planning, and health economic assessments, as well as cost-benefit analyses.

## Figures and Tables

**Table 1:** Participant characteristics (median and 1st–3rd quartile) (*n* = 1661).

Sex	Age Years	Height cm	Weight kg	BMI kg·m^−1^	Distance m	Duration min	Velocity km·h^−1^	Cycling Environment *
Male (*n* = 562)	47 38–57	180 176–185	78 72–84	23.9 22.4–25.4	7794 4594–12,790	25 19–40	17.9 14.7–21.3	I = 94
								I–S = 277
								S = 191
Female (*n* = 1099)	47 39–55	168 164–172	64 59–70	22.6 21.0–24.4	4900 3000–8050	20 15–33	14.0 11.6–16.5	I = 242
								I–S = 388
								S = 469

Note: * Number of cyclists in: I = inner urban; I–S = inner urban–suburban; S = suburban.

**Table 2:** Expected and detected distributions of last digit categories in male and female commuter cyclists (*n* = 1661). The absolute number in each group is also indicated.

Last Digits in Self-Reported Cycle Trip Durations	Males		Females	
	Expected Distribution % (Number)	Detected Distribution % (Number)	Expected Distribution % (Number)	Detected Distribution % (Number)
0 or 5	20% (112)	69% (387)	20% (220)	75% (819)
1–4 or 6–9	80% (450)	31% (175)	80% (879)	25% (280)

**Table 3:** Correlation coefficients between continuous outcome and predictor variables (*n* = 1558).

Variable	Velocity	Distance	Duration	Age	Weight	BMI
Velocity	-					
Distance	0.67 ***	-				
Duration	0.32 ***	0.89 ***	-			
Age	−0.21 ***	−0.07 **	0.03 n.s.	-		
Weight	0.24 ***	0.19 ***	0.09 ***	0.07 **	-	
BMI	0.02 n.s.	0.06 *	0.06 *	0.16 ***	0.76 ***	-

Note: n.s. = not significant, * = *p* <0.05, ** = *p* < 0.01, *** = *p* < 0.001.

**Table 4:** Multiple regression analyses of the relation between cycle commuting speed and distance, as well as other predictors. All calculations are based on data coupled to self-reported durations of ≤50 min (*n* = 1558).

Model 1 R^2^ = 0.56										
Outcome Variable		Predictor Variables								
	Cycling Velocity (km·h^−1^)		Distance (km)	Sex (0 = Female; 1 = Male)	Age (years)	Weight (kg)	BMI (kg·m^−2^)	Last Digit in Duration Self-Reports (0 = 1–4 or 6–9; 1 = 0 or 5)	Cycling Environment (0 = Suburban; 1 = Suburban–Inner Urban)	Cycling Environment (0 = Suburban; 1 = Inner Urban)
**y-intercept**	16.2	**unstandardized regression coefficient B**	0.64	1.69	−0.066	0.036	−0.15	−1.66	−0.35	−0.65
**95% CI**	14.8–17.6	**95% confidence interval**	0.60–0.68	1.26–2.13	−0.079–−0.053	0.008–0.064	−0.25–−0.06	−1.98–−1.34	−0.69–−0.02	−1.03–−0.26
***p*-value**	0.000	***p*-value**	0.000	0.000	0.000	0.011	0.001	0.000	0.038	0.001
		**partial correlation**	0.62	0.19	−0.24	0.06	0.08	−0.25	−0.05	−0.08

**Table 5:** Multiple regression analyses of the relation between cycle commuting speed and duration as well as other predictors. All calculations are based on self-reported durations of ≤50 min (*n* = 1558).

Model 2 R^2^ = 0.34										
Outcome Variable		Predictor Variables								
	Cycling Velocity (km·h^−1^)		Duration (min)	Sex (0 = Female; 1 = Male)	Age (years)	Weight (kg)	BMI (kg·m^−2^)	Last Digit in Duration Self-Reports (0 = 1–4 or 6–9; 1 = 0 or 5)	Cycling Environment (0 = Suburban; 1 = Suburban–Inner Urban)	Cycling Environment (0 = Suburban; 1 = Inner Urban)
**y-intercept**	18.3	**unstandardized regression coefficient B**	0.096	2.67	−0.084	0.051	−0.20	−1.49	0.33	−1.59
**95% CI**	16.6–20.0	**95% confidence interval**	0.079–0.113	2.14–3.19	−0.100–−0.068	0.017–0.086	−0.31–−0.08	−1.89–−1.09	−0.08–0.75	−2.06–−1.12
***p*-value**	0.000	***p*-value**	0.000	0.000	0.000	0.003	0.001	0.000	0.114	0.000
		**partial correlation**	0.27	0.24	−0.25	0.08	−0.09	−0.18	0.04	−0.17

## References

[1] Statens vegvesen Vegdirektoratet (2014). Veiledning. Konsekvensanalyser. Håndbok V712.

[2] Kahlmeier S., Kelly P., Foster C., Götschi T., Cavill N., Dinsdale H., Woodcock J., Schweizer C., Rutter H., Lieb C., World Health Organization Regional Office for Europe (2014). Health Economic Assessment Tool (HEAT) for Walking and Cycling.

[3] Ainsworth B.E., Haskell W.L., Whitt M.C., Irwin M.L., Swartz A., Strath S.J., O’Brien W.L., Bassett D.R., Schmitz K.H., Emplaincourt P.O. (2000). Compendium of Physical Activities: An update of activity codes and MET intensities. Med. Sci. Sports Exerc..

[4] Allen D., Rouphail N., Hummer J., Milazzo J. (1998). Operational Analysis of Uninterrupted Bicycle Facilities. Transp. Res. Rec..

[5] El-Geneidy A.M., Krizek K.J., Iacono M. Predicting bicycle travel speeds along different facilities using GPS data: A proof of concept model. Proceedings of the 86th Annual Meeting of the Transportation Research Board.

[6] Coluccia E., Louse G. (2004). Gender differences in spatial orientation: A review. J. Environ. Psychol..

[7] Crompton A., Brown F. (2006). Distance estimation in a small-scale environment. Environ. Behav..

[8] Gärling T., Loukopoulos P., Allen G.L.E. (2007). Choice of driving versus walking related to cognitive distance. Applied Spatial Cognition: From Research to Cognitive Technology.

[9] Stigell E., Schantz P. (2011). Methods for Determining Route Distances in Active Commuting—Their Validity and Reproducibility. J. Transp. Geogr..

[10] Schantz P., Stigell E. (2009). A criterion method for measuring route distance in physically active commuting. Med. Sci. Sports Exerc..

[11] Rietveld P. (2001). Rounding of Arrival and Departure Times in Travel Surveys.

[12] Kelly P., Krenn P., Titze S., Stopher P., Foster C. (2013). Quantifying the difference between self-reported and global positioning systems-measured journey durations: A systematic review. Transp. Rev. Trans. Transdiscipl. J..

[13] Kelly P. (2013). Assessing the Utility of Wearable Cameras in the Measurement of Walking and Cycling. Ph.D. Thesis.

[14] Stigell E., Schantz P. (2015). Active commuting behaviours in a nordic metropolitan setting in relation to modality, gender, and health recommendations. Int. J. Environ. Res. Public Health..

[15] Åstrand P.-O., Ryhming I. (1954). A nomogram for calculation of aerobic capacity (physical fitness) from pulse rate during sub-maximal work. J. Appl. Physiol..

[16] Atkinson G., Davison R., Jeukendrup A., Passfield L. (2003). Science and cycling: Current knowledge and future directions for research. J. Sports Sci..

[17] Faria E.W., Parker D.L., Faria I.E. (2005). The science of cycling: Physiology and training—Part 1. Sports Med..

[18] Åstrand I. (1960). Aerobic work capacity in men and women with special reference to age. Acta Physiol. Scand..

[19] Åstrand P.-O., Rodahl K. (1970). Textbook of Work Physiology. Physiological Bases of Exercise.

[20] Swain D.P. (1994). The influence of body mass in endurance bicycling. Med. Sci. Sports Exerc..

[21] Åstrand I., Åstrand P.-O., Stunkard A. (1960). Oxygen intake of obese individuals during work on a bicycle ergometer. Acta Physiol. Scand..

[22] Berry M.J., Storsteen J.A., Woodard C.M. (1993). Effects of body mass on exercise efficiency and VO2 during steady-state cycling. Med. Sci. Sports Exerc..

[23] Wahlgren L., Schantz P. (2011). Bikeability and methodological issues using the active commuting route environment scale (ACRES) in a metropolitan setting. BMC Med. Res. Methodol..

[24] The Swedish School for Sport and Health Sciences, GIH, Stockholm, Sweden: The Research Project Physically Active Commuting in Greater Stockholm (PACS). http://www.gih.se/pacs.

[25] Johansson C., Lövenheim B., Schantz P., Wahlgren L., Almström P., Markstedt A., Strömgren M., Forsberg B., Sommar J.N. (2017). Impacts on air pollution and health by changing commuting from car to bicycle. Sci. Total Environ..

[26] Wahlgren L., Stigell E., Schantz P. (2010). The active commuting route environment scale (ACRES): Development and evaluation. Int. J. Behav. Nutr. Phys. Act..

[27] Stockholm Office of Research and Statistics (2006). Statistical Year-Book of Stockholm 2006. Area and Population Density by City District.

[28] Field A. (2005). Discovering Statistics Using SPSS.

[29] Åstrand P.-O. (1952). Experimental Studies of Physical Working Capacity in Relation to Sex and Age.

